# Affected albumin endocytosis as a new neurotoxicity mechanism of amyloid beta

**DOI:** 10.3934/Neuroscience.2020021

**Published:** 2020-09-23

**Authors:** Lourdes A. Vega Rasgado, Arantxa Tabernero Urbieta, José María Medina Jiménez

**Affiliations:** 1 Laboratorio de Neuroquímica, Departamento de Bioquímica, Escuela Nacional de Ciencias Biológicas, Instituto Politécnico Nacional, Ciudad de México, México; 2 Instituto de Neurociencias de Castilla y León (INCYL), c/Pintor Fernando Gallego 1, 37007 Salamanca, Spain

**Keywords:** Alzheimer's disease, amyloid-β, albumin, endocytosis, neuronal dead

## Abstract

Senile plaques, a hallmark of Alzheimer's disease, are composed by Amyloid-Beta (Aβ). Aβ 25-35 toxicity is caused mainly by increasing reactive oxygen species (ROS), which is reversed by albumin preventing Aβ internalization. In addition, key cellular processes and basic cell functions require of endocytosis, particularly relevant in neurons. To understand the protective effect of albumin and the toxicity mechanism of Aβ, the need of albumin uptake for neurons protection as well as the possible influence of Aβ on albumin endocytosis were investigated. With this aim the influence of lectin from soybeans (LEC), which prevents albumin endocytosis, on the effects of Aβ 25-35 on cellular morphology and viability, ROS generation and Aβ uptake with and without albumin in neurons in primary culture was investigated. Influence of Aβ on albumin endocytosis was studied using FITC-labelled albumin. LEC did not modify Aβ effects with or without albumin on neuronal morphology, but increased cell viability. LEC increased ROS generation with and without Aβ in the same magnitude. Diminished Aβ internalization observed with albumin was not affected by LEC. In presence of Aβ albumin is internalized, but endosomes did not deliver their cargo to the lysosomes for degradation. It is concluded that formation of Aβ-albumin complex does not require of albumin internalization, thus is extracellular. Aβ affects albumin endocytosis preventing late endosomes and lysosomes degradation, probably caused by changes in albumin structure or deregulation in vesicular transport. Considering the consequences such as its osmotic effects, the inability to exert its antioxidant properties, its effects on neuronal plasticity and excitability albumin affected endocytosis induced by Aβ is proposed as a new physiopathology mechanism in AD. It is hypothesized that there is critical intraneuronal level above which albumin becomes toxic.

## 1. Introduction

Key cellular processes such as nutrient uptake, signal transduction or membrane recycling require of endocytosis. Basic cell functions such as sorting, signaling, recycling and degradative functions require positioning, regulation, and trafficking of endocytic organelles. Endocytosis is particularly relevant in neurons since it has unique demands because of the extremely long distance spanned by a neuron. Besides, it must be remembered that once neurotransmitters were released, the synaptic vesicle membrane and proteins are internalized by endocytosis. Thus, endocytosis of synaptic vesicles is important for maintaining synaptic vesicle pools.

In neurons, solutes such as lipids, ligands and proteins among some others penetrate cells by different endocytic pathways. All endocytic processes begin with the uptake of the structures or molecules and the formation of primary endocytic vesicles [1]. Then, vesicles are uncoated and form early endosomes by fusion between each other as well as with preexisting endosomes. Finally, endosomes contents can be released in several ways [2].

One of the main types of endocytosis is clathrin-dependent, in which vesicles are covered by this protein [3]. This allows neurons to respond quickly to different external stimuli [4],[5], for example during axonal growing [6].

There is also clathrin-independent endocytosis, which include caveolin-mediated endocytosis [4],[5]. There is still so much to learn about the events modulated by caveolin in neurons, but this protein could play an important role in processes such as neuronal migration and plasticity [6]. This idea is supported by the fact that endosome function is altered in different neurodegenerative axonal neuropathies [7].

One of the most important neurodegenerative disorders, considered as a primary dementia, causing 46.8 million people worldwide in 2015, is Alzheimer disease (AD). There are two neuropathological hallmarks of AD, the formation of amyloid plaques and neurofibrillary tangles. Amyloid plaques are the result of extracellular accumulation of fibrillar amyloid-β (Aβ) [8]. Aβ peptides are considered to play a key role in AD research because genetics studies have indicated that they are involved in all known forms of familial AD [9]. However, the molecular mechanism of Aβ neurotoxicity has not been elucidated yet.

The more frequent Aβ peptides found in amyloid plaques are Aβ 40 and Aβ 42, which are formed from amyloid-β-protein precursor by β and γ secretase [10]. Besides enzymatic catalysis, recently it has been reported that these peptides can be detached from the plaques by a spontaneous mechanism [11]. The shorter Aβ peptide that remains toxic, Aβ 25-35, has also been found in the brain of AD patients, presumably coming from Aβ 40 cleavage [12]. This suggests that Aβ 25-35 together with Aβ 40 and Aβ 42 can play a role in the pathogenesis of AD.

In body fluids such as cerebrospinal fluid (CSF) and blood, Aβ is transported as Aβ-albumin complex [13],[14]. Albumin is the most abundant protein in the human body, including both plasma and CSF, with a very important role as volume expander and transporter. Serum albumin is not only the way to transport Aβ, it has very important functions in the central nervous system (CNS). Albumin has different roles in developing brain, where it reaches high concentrations. Neurons [15],[16] as well as astrocytes [17],[18] perform active uptake of albumin. Albumin also participates in regulation of brain cells metabolism [19],[20]. In absence of external neurotrophic factors albumin prevent neuronal apoptosis in cells in primary culture by increasing the synthesis and release of glutamate, keeping their differentiation program [21]. It is also considered that serum albumin plays an important role in Aβ clearance and elimination in kidney [22]–[25].

Instead of its high plasma concentration brain albumin levels are low, about 3 µM [26], maybe because an active uptake is required to cross through the blood-brain barrier (BBB). The BBB precisely controls the entry of blood components, including plasma proteins, ions, red blood cells and leukocytes into the CNS, as well as the elimination of toxic molecules to the blood [27]–[29]. BBB disruption allows influx into the brain of proteins, cells, neurotoxic blood-derived debris, microbial pathogens, etc. Thus, the disturbance of proper BBB functioning is increasingly recognized as a potential contributor in several neurological disease pathogenesis, including AD [30]–[32]. As a fact, in AD patients brain albumin levels are increased, and CSF-albumin index is considered a measure of disease progression over one year [33].

It has been previously shown that morphological damage and neuronal toxicity caused by Aβ 25-35 by increasing reactive-oxygen-species (ROS) levels, were counteracted by albumin [34]. Aβ 25-35 has also deep effects on neuronal plasticity that were partially reversed by albumin, as demonstrated by the decreased expression of Growth Associated Protein 43 (GAP-43) and Microtubules Associated Protein-2 (MAP-2), associated proteins with axonal and dendritic growth respectively, which were raised by albumin. Association with serum albumin may prevent Aβ polymerization [35], and it is proposed that albumin exerts its protective effects by binding to Aβ in an equimolecular way, which prevents heterodimer (Aβ-BSA) entry into the neurons [34]. Besides, albumin is the main extracellular antioxidant, which may also explain its protective properties against Aβ.

Considering that endosomal traffic is particularly important in neurons [2], the different roles of albumin in neurons and its protective properties against Aβ and that albumin entrance into the neurons is mediated by endocytosis, affected albumin endocytosis was investigated as a possible new toxicity mechanism of Aβ. With this aim soybean lectin (LEC), an agglutinin which reacts specifically with BSA promoting its precipitation, was employed. After endocytosis, albumin is transported to late endosomes and lysosomes for degradation [36],[37]. Hence, how degradation of BSA is modified by Aβ in neurons in primary was also studied.

## 2. Materials and methods

### 2.1. Animals

All experiments were performed according to the rules for the protection of animals used in research and other scientific purposes in the European Union.

Albino Wistar rats fed *ad libitum* on a stock laboratory diet (49.8% carbohydrates, 23.5% protein, 3.7% fat, 5.5% minerals and added vitamins and amino acids; w/w) were used for the experiments. The animals were maintained on a 12 h light-dark cycle. Females with a mean weight of 250 g were caged with males overnight and conception was considered to occur at 01:00; this was verified the following morning by the presence of spermatozoa in the vaginal smears. To prepare neurons in primary culture, fetuses at 17.5 days of gestation were delivered by rapid hysterectomy after cervical dislocation of the mother.

### 2.2. Reagents

Dulbeco's modified Eagle's medium (DMEM), penicillin, streptomycin, poly-L-lysine, cytosine arabinoside, bovine serum albumin (BSA; fatty acid-free and dialyzed before use) and lectin Glycine max (Soybean lectin; LEC) were purchased from Sigma-Aldrich Chemical Co. (Madrid, Spain). Aβ 25-35 was obtained from Bachem (Torrance, California, USA) and was dissolved in deionized water. Fetal calf serum (FCS) was obtained from Serva Boehringer Ingelheim (Heidelberg, Germany). Fluorogenic 2′,7′-dichlorodihydrofluorescein-diacetate probe (DCFH-DA) was acquired from Molecular Probes (Eugene, OR, USA). Antibody against Aβ 25-35 was obtained from Biosurce (Biosurce International, Inc, USA), and secondary antibody antirabbit Cyanine 3 (Cy3) was purchased from Sigma-Aldrich Chemical Co. Albumin conjugated with fluorescein isothiocyanate (FITC-BSA) was purchased from Sigma-Aldrich Chemical Co. The medium to preserve and visualization of fluorescence was "SlowFade Gold Antifade Reagent” from Invitrogen.

Standard analytical grade laboratory reagents were obtained from Merck (Dramstadt, Germany) or Sigma-Aldrich Chemical Co.

### 2.3. Neuronal cultures

Cells were cultured essentially as described by Tabernero et al. [38]. Briefly, once animals were decapitated their brains were immediately excised. After removing the meninges and blood vessels, the forebrain was placed in Earle's balanced solutions containing 20 µg/ml DNase and 0.3% (w/v) BSA. The tissue was minced, washed, centrifuged at 500x g for 2 min and incubated in 0.025% trypsin (Type III) and 60 µg/ml DNase I for 15 min at 37 °C. Trypsinization was halted by the addition of DMEM supplemented with 10% FCS. The tissue was dissociated by passing it gently 4 to 8 times through a siliconized Pasteur pipette and the supernatant was recovered. This operation was repeated 3 or 4 times and the resulting cell suspension was centrifuged at 500x g for 5 min. The cells were resuspended in DMEM containing 10% FCS and counted in a Neubauer chamber (test for the exclusion of trypan blue dye was used to indicate cell viability). Once diluted in DMEM supplemented with 10% FCS, 50 U/ml penicillin, 37.5 U/ml streptomycin, and 25 mM KCl, cells were plated on 3.5 cm or 6.0 cm diameter Petri dishes coated with 10 µg/ml of poly-L-lysine at a density of 1.5 × 10^5^ cells/cm^2^. One day after plating, 10 µM cytosine arabinoside was added to avoid glial cell proliferation.

### 2.4. Neuronal Survival and morphology

For survival experiments, after the corresponding treatment neuronal viability was determined by the 3-(4,5-Dimethylthiazol-2-Yl)-2,5-Diphenyltetrazolium Bromide (MTT) reduction assay [39]. This assay was selected after comparing results with other methods such as counting cells stained with Hoechst and propidium iodide and lactate dehydrogenase activity, in order to corroborate Aβ 25-35 toxicity. This method is based on the ability of active mitochondrial dehydrogenases to convert MTT (dissolved in PBS, 5 mg/ml) to water-insoluble purple formazan crystals. MTT was diluted 1:10 in DMEM and added to the cells. At the end of the incubation period (75 min in darkness), the medium with MTT was replaced by dimethyl sulfoxide and gently shaken for 10 min in darkness. Then, 100 µl of lysate was transferred to a 96-well plate and the absorbance of the dye was measured at a wavelength of 570 nm. Data are presented as percentages of absorbance compared with control cells. Experiments were repeated at least 5 times with 3 wells per condition.

For morphological studies phase contrast photographs were taken and labeled according to the culture conditions.

#### 2.4.1. Influence of lectin Glycine max (Soybean lectin) on amyloid beta effect on neuronal survival and morphology with and without albumin

Three days after plating cultured neurons in DMEM supplemented with FCS 10% (v/v) were deprived from serum, keeping them in Hank's medium. Cells were preincubated with and without LEC (50 µg/ml) for 30 minutes and then exposed to Aβ 25-35 (30 µM) with and without BSA (30 µM). 24 hours after corresponding treatment, neuronal viability and morphology were investigated.

### 2.5. Quantification of reactive oxygen species

Production of ROS was measured using DCFH-DA, which is intracellularly deacetylated to 2′,7′-dichlorodihydrofluorescein (DCFH) and then converted to the fluorescent oxidized compound 2′,7′-dichlorofluorescein (DCF) by hydrogen peroxide [40].

#### 2.5.1. Influence of lectin Glycine max (Soybean lectin) on reactive oxygen species generation induced by amyloid beta with and without albumin

Neurons were cultured in DMEM with 10% of FCS (v/v) for three days. Then, cells were maintained in a serum free medium (Hank's medium, pH = 7.4) and preincubated with and without LEC (50 µg/ml) for 30 minutes. Coming up next neurons were exposed to Aβ 25-35 (30 µM) with and without BSA (30 µM). ROS were quantified 24 hours after described treatments.

### 2.6. Immunocytochemistry for Aβ

Immunocytochemistry for Aβ was essentially carried out as described by Tabernero et al. [41]. After the corresponding treatment, neurons were fixed in 4% paraformaldehyde for 30 min and after a wash with PBS they were permeabilized with 0.25% Triton X-100 for 1 hour. Cells were then incubated with antibody against Aβ 1:2000 overnight. Then, cells were washed with PBS and incubated with antirabbit Cy3 1:1000 in the darkness for 1 hour. After through wash with PBS, fluorescence photographs were taken with a confocal microscope. Different fields were analyzed in each Petri dish from at least 5 experiments. Fluorescence intensity was analyzed using Scion Image software (Wayne Rasband, National Institute of Health, USA).

#### 2.6.1. Influence of lectin Glycine max (Soybean lectin) on amyloid beta internalization in neurons with and without albumin

Neurons were cultured in DMEM supplemented with FCS 10% (v/v) for three days. Then, cells were deprived from serum (keeping them in Hank's medium) and were preincubated with and without LEC (50 µg/ml) for 30 minutes. After that Aβ 25-35 (30 µM) was added with and without BSA (30 µM). 24 hours later neurons were fixed with paraformaldehyde in order to perform the immunocytochemical study as described before.

### 2.7. Albumin uptake

For visualization of this process the marker FITC-BSA 2% was used. After corresponding treatment with FITC-BSA, neurons were washed several times with PBS solution and fixed in 4% paraformaldehyde for 30 min in darkness. Then, cells were washed several times with PBS and the protector medium of fluorescence was added. Photographs were taken with a confocal microscope and different fields were analyzed in each Petri dish from at least 5 experiments. Fluorescence intensity was analyzed using Scion Image software (Wayne Rasband, National Institute of Health, USA).

#### 2.7.1. Influence of amyloid beta at different times on albumin endocytosis in neurons

After three days of being cultured in DMEM supplemented with FCS 10% (v/v), neurons were maintained in a serum free medium (Hank's medium, pH = 7.4) and FITC-BSA was added for 15, 30 or 45 minutes in presence as well as in absence of Aβ 25-35 (30 µM). After corresponding time of treatment, cells were fixed in 4% paraformaldehyde and the immunocytochemical studies of albumin uptake were carried out as described above.

### 2.8. Statistical analysis

All results are expressed as the mean ± standard error of the mean (SEM) of at least five determinations (n ≥ 5). Neuronal viability and relative fluorescence levels, expressed as a percentage respect to the control group, were compared between groups using independent factorial analysis of variance (ANOVA) or repeated-measures ANOVA. For Post-Hoc analysis Tukey's multiple comparison test was employed. Graph Pad Prism version 5.0 software (GraphPad Software, Inc, La Jolla, CA, USA) was used, and different characters were used to indicate statistically different groups as compared with the control. Thus, a group is identified with a character, and if this group is statistically different to any other a different character is employed. If there is not statistical difference between those groups, the same character is used (Significant p ≤ 0.05).

## 3. Results

### 3.1. Influence of lectin Glycine max (Soybean lectin) on amyloid beta effect on neuronal survival and morphology with and without albumin

In order to investigate if albumin uptake was required for its protective effect against Aβ, albumin endocytosis was impaired with LEC and its protective effect was compared with that of albumin with “free” endocytosis. Inhibition of interaction between LEC and cell surfaces by N-acetyl-D-galactosamine is well known since long time ago [42]. Albumin internalization in neurons is mediated by a receptor, which could be described as a glycoprotein containing N-acetyl-galactosaminyl or galactosaminyl residues. Thus, albumin endocytosis is inhibited by LEC [43],[44]. Because of the former, LEC was used as a tool to study the influence of albumin internalization on its protective effect. Specifically, the influence of LEC on Aβ effect on neuronal morphology and viability in presence as well as in absence of albumin was investigated.

**Figure 1.**
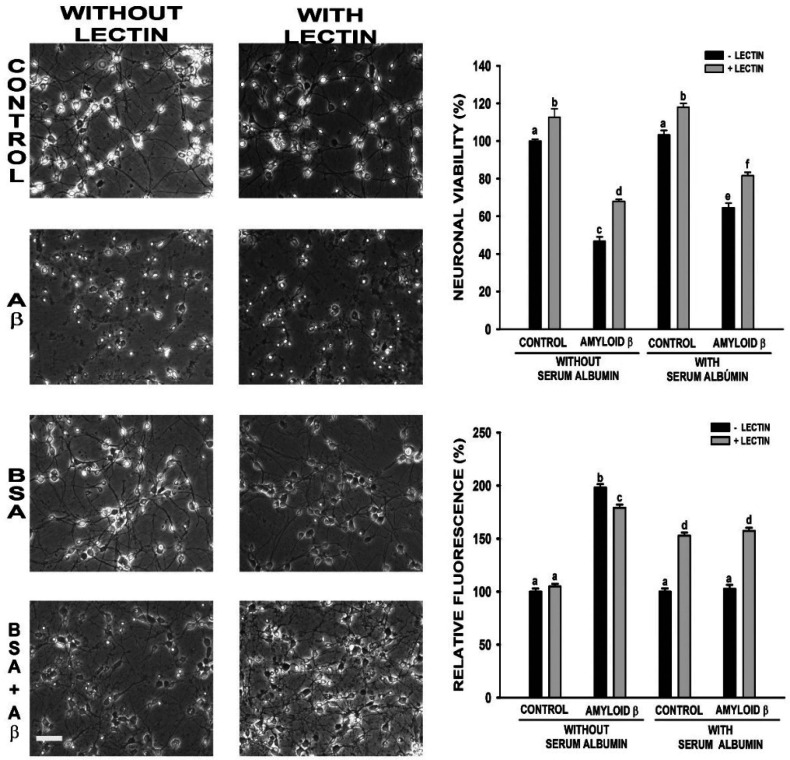
Influence of lectin *Glycine* *max* (Soybean lectin) on effects of albumin (BSA) on cellular morphology, viability and reactive oxygen species (ROS) generation of neurons in primary culture exposed to amyloid beta 25-35 (Aβ).

About neuronal morphology (Figure 1, left), it can be appreciated that Aβ 25-35 damaged cell morphology, but it was improved by albumin. LEC did not modify effect of Aβ 25-35 on morphology neither with nor without albumin, which means that LEC did not reverse the protective effect of albumin against Aβ 25-35 effects on neuronal morphology.

Besides described morphological effects, the toxic effect of Aβ 25-35 and the protective effect of albumin were verified by measuring their effects on neuronal survival (Figure 1 right up). Aβ 25-35 decreased cell viability by about 50%, which is in coincidence with results observed in LDH and account of stained cell methods (data not shown). Albumin increased neuronal survival, which goes from 50 to 70%, confirming its protective effect. The treatment with LEC did not counteract the albumin protection against Aβ. On the contrary, it increased neuronal survival in all cases (Figure 1 right up).

### 3.2. Influence of lectin Glycine max (Soybean lectin) on reactive oxygen species generation induced by amyloid beta with and without albumin

Considering that oxidative damage brought about by an increased ROS generation is a very important event in the toxicity of Aβ 25-35 in cultured neurons, which was not observed in presence of albumin [34], it was considered particularly relevant to investigate the influence of LEC on this parameter. Results, expressed as % respect to the corresponding control and normalized by neuronal viability (Figure 1 right down), indicate that LEC did not modify essentially ROS generation induced by Aβ 25-35 in absence of albumin. In contrast, LEC significantly increased ROS generation either with or without Aβ 25-35 about the same magnitude (55%) in presence of albumin.

Neurons were cultured in DMEM with 10% (v/v) FCS for 3 days. Then, they were serum-deprived and 30 µM Aβ 25-35 was added in for 24 hours with and without 30 µM BSA, in presence as well as in absence of LEC (50 µg/ml). Phase-contrast photographs were taken and labeled according to the culture conditions (Bar = 100 µm, left panel). Cellular viability was determined by the MTT reduction method (right panel up). ROS were quantified using DCFH (right panel down). Results are presented as the mean ± SEM and were expressed as percentage of the respective controls. An independent ANOVA test was applied, and different characters were used to indicate statistically different groups as compared with the control (N ≥ 6, observations each experiment = 4, total degree of freedom 42, F = 3.037, p ≤ 0.05, Post-hoc analysis Newman-Keuls test).

### 3.3. Influence of lectin Glycine max (Soybean lectin) on amyloid beta internalization in neurons in primary culture, with and without albumin

**Figure 2.**
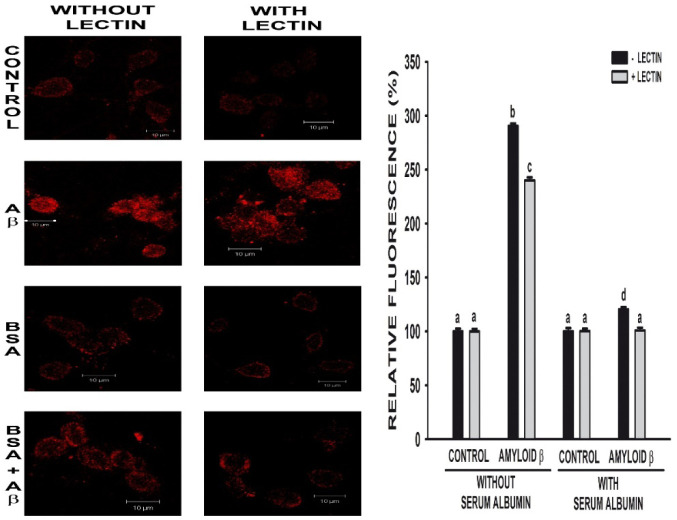
Effect of lectin *Glycine* *max* (Soybean lectin) on amyloid beta (Aβ) internalization in neurons in primary culture in presence as well as in absence of albumin (BSA).

Figure 2 shows internalization of Aβ 25-35 and how albumin decreased its entry into neurons. These results are not different from observed in presence of LEC, which indicates that the impediment of albumin endocytosis did not modify Aβ 25-35 income into neurons nor effect of albumin on Aβ 25-35 internalization.

Neurons were cultured in DMEM with 10% (v/v) FCS for 3 days. Then, they were serum-deprived and preincubated with and without LEC (50 µg/ml) for 30 minutes. Thereafter, 30 µM Aβ 25-35 was added in for 24 hours with and without 30 µM BSA. Cells were fixed and processed for Aβ immunocytochemistry, as described in Materials and Methods. Fluorescence photomicrographs were taken with a confocal microscope (bar = 10 µm) and were quantified with “Scion image for Windows” software. Results are presented as the mean ± SEM and were expressed as a percentage of that obtained in the control group. Results are expressed as percentage of the respective controls. An independent factorial ANOVA test was applied, and different characters were used to indicate statistically different groups compared with the control (N ≥ 6, observations each experiment = 4, total degree of freedom 42, F = 3.051, p ≤ 0.05, Post-hoc analysis Newman-Keuls test).

### 3.4. Influence of amyloid beta at different times on albumin endocytosis in neurons

Because of the importance of endocytosis in neurons, the key role of albumin in the brain and its close relation with AD, the influence of Aβ 25-35 in albumin uptake was investigated.

**Figure 3.**
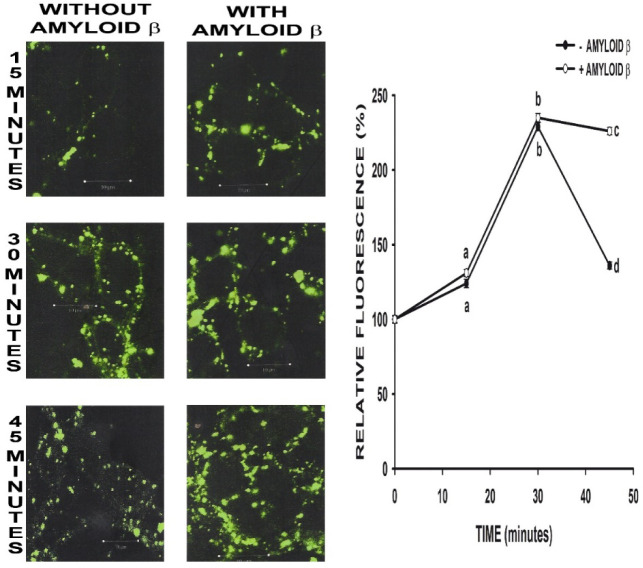
Albumin (BSA) internalization at different times in neurons in primary culture in presence as well as in absence of amyloid beta (Aβ).

Images on figure 3 show the progressive internalization of albumin into neurons and the formation of small vesicles. After 45 minutes, described vesicles were dispersed and fluorescence could be appreciated in the whole cell, which indicates intracellular degradation of albumin vesicles. However, when Aβ 25-35 was added at the same incubation time BSA remained retained in vesicles, showing a peripheral distribution.

Neurons were cultured in DMEM with 10% (v/v) FCS for 3 days. Then, they were serum-deprived and a solution of BSA conjugated with FITC-BSA (2% p/v) was added in with and without 30 µM Aβ 25-35. After the corresponding time of treatment, cells were fixed and processed for Aβ immunocytochemistry as described in Materials and Methods. Fluorescence photomicrographs were taken with a confocal microscope (bar = 10 µm) and were quantified with “Scion image for Windows” software. Results are presented as the mean ± SEM and were expressed as a percentage of that obtained in the control group. Results are expressed as percentage of the respective controls. A repeated-measured ANOVA test was applied, and different characters were used to indicate statistically different groups compared with the control (N ≥ 6, observations each experiment = 4, total degree of freedom 20, F = 3.196, p ≤ 0.05, Post-hoc analysis Tukey's multiple comparison test).

## 4. Discussion

The main reason to select Aβ 25-35 for these experiments was its high toxicity in cell cultures [11], which is considered a result of fibrillar aggregates formation [45],[46]. The use of Aβ 25-35 in studies of cellular dysfunction has provided data indicating that the peptide is indeed responsible for multiple disturbances to cellular integrity. It has also been described how Aβ 25-35 induces oxidative stress, disturbs calcium homeostasis and damage multiple enzymes [34],[47]. Aβ 25-35 is considered the biologically active region of Aβ 40 or 42, keeping similar properties and toxicity that observed in Aβ 42 and Aβ 40 [48]. Specifically, Aβ 25-35 toxicity has been described in hippocampus neurons cultures [11] as well as in hippocampus slices [49], resulting quite similar to that observed in Aβ 42 and Aβ 40 [50]. Even more, before informing the toxic effect of Aβ 25-35 and the protective effect of albumin, parallel studies were performed with Aβ 40 with similar results [34]. Hence, Aβ 25-35 was used thereafter.

Neuronal morphology was not appreciably modified by LEC in any of the different treatments performed (Figure 1). The former indicates that LEC has not toxic effects itself and means that albumin internalization is not required for its protective effect. It has been proposed that albumin protective effect could be explained by the formation of Aβ-Albumin complex [34]. These results suggest that such complex would be extraneuronal, which would explain that albumin uptake is not necessary to protect against toxic effects of Aβ 25-35.

Avoiding albumin endocytosis with LEC [43] increased neuronal viability in cells treated with BSA, even in presence of Aβ 25-35. Cellular uptake of Aβ 1-40 and Aβ 1-42 occurs exclusively via endocytosis, however there are interesting differences between the two Aβ variants [51]. Thus, the decrease on neuronal mortality induced by Aβ 25-35 in presence of LEC may indicate that Aβ 25-35 is also endocytosed into the cells, and LEC prevents not only albumin but also partially Aβ 25-35 internalization. This hypothesis seems to be supported by immunocytochemical studies, which show a decrease on Aβ 25-35 internalization when albumin endocytosis is inhibited (Figure 2).

Albumin is considered the main extracellular antioxidant. Once into the neurons, due to its concentration and molecular properties albumin becomes a target for oxidation reactions. Furthermore, albumin binds metals and heme thereby limiting their ability to produce ROS. Hence, if albumin is not endocytosed, then it could not exert its antioxidant properties. This may be the reason of the increase of ROS generation when LEC is added, regardless of the presence of Aβ 25-35.

Considering that endocytosis is particularly important in neurons, the key role of albumin and that it is endocytosed in these cells, the influence of Aβ 25-35 specifically on the endocytic process of albumin was also investigated. In figure 3 can be appreciated that albumin endocytic vesicles were not degraded and shown a peripheral distribution, which indicate that the process of late endosomes and lysosomes degradation is affected by Aβ 25-35. The lack of lysosomes degradation may be due to a conformational change produced by Aβ on tertiary structure of albumin. This would lead to the anchorage of the protein to the neuronal surface by the hydrophobic end of Aβ. That is in coincidence with the proposal of changes on albumin structure induced by the presence of different molecules, specially observed in albumin of patients with AD [52].

Degradative metabolism is essential to the maintenance of cellular homeostasis, hence the misdirection of signaling ligands or other important materials into degrading pathways would be detrimental to the cell. When this critical sorting event is altered, there is not a proper retention and recycling of endocytosed materials locally. Their shipment to more distant parts of the cell for signaling or degradation is also disturbed. In this case, the retention of albumin in vesicles cause its intraneuronal increase. Albumin into the neurons is not an inert bystander. On the contrary, *in vitro* studies indicate that albumin administration significantly increased neuronal damage and dead in a dose and time-dependent manner [53]. Such deleterious actions may be explained by the different albumin effects. As an example, the osmotic effects of albumin injection into the neostriatum in one hemisphere resulted in a greater lesion volume, compared to injection of saline into the other hemisphere in the rat [54]. Besides extravasated albumin could induce proinflammatory events, such as the expression of proinflammatory cytokines [55].

Another example of the possible consequences of albumin retention into endocytic vesicles, is that their cargo would not be properly delivered. Since albumin is the main transporter of different substances such as fatty acids, intraneuronal levels of such compounds may decrease or modify its subcellular localization. This could be related with the toxic effects of Aβ 25-35, which has deep effects on neuronal plasticity, as demonstrated by this peptide effects on the expression of markers of axonal as well as dendritic growth. Specifically, the expression of GAP-43 and MAP-2, associated proteins with axonal and dendritic growth respectively, were diminished by Aβ 25-35 [34].

Although GAP-43 protein is located into the inner surface of the plasma membrane of growing axons it is a hydrophilic protein, which is not usual for membrane associated proteins. Analysis has revealed a short hydrophobic amino acid sequence segment which functions to anchor the GAP-43 protein on the cytoplasmic side of the presynaptic plasma membrane [56],[57]. The addition of palmitate to cysteine residues enhances the hydrophobicity of proteins, thus its membrane association. Because of the rapid turnover and the variation of palmytoylation levels with extracellular stimuli [58]–[60], it is considered that addition or removal of palmitate regulates the activity of proteins. In this case, a reduction in the palmitoylation of GAP-43 is sufficient to stop advancing axons during critical remodeling periods and even to decline the availability of this protein [61].

The importance of the palmitoylated domains to protein function is demonstrated. Its effects could be attributable to the absence of palmitate, to the substituted amino acid residue or to an altered subcellular localization. In any case albumin, the transporter of palmitate, would play a key role in the variation of palmitoylation levels, which regulates proteins activity [58]–[60]. Thus, changes in GAP-43 expression caused by Aβ 25-35 may be the result of altered endocytic process of albumin.

Another important effect of albumin is an increase on neurons excitability by increasing the synthesis of glutamate [21]. Albumin induce the expression of IL-1β, which was found to be associated with inducing excitatory postsynaptic currents and glutamate cytotoxic neuronal damage [62]. Glutamatergic deregulation may lead to brain cell death or dysfunction through destruction of synaptic function and plasticity, promotion of neuroinflammation and the release of Aβ and tau [63],[64]. An example of the participation of glutamatergic transmission on AD physiopathology, indirectly regulated by albumin, is the inhibition of dementia symptoms for a limited period by the use of antagonist of the N-methyl-D-aspartate receptors or the use of glutamate transporters in patients with mild to moderate AD, which trials are now at days in phase 2 and 3 [65],[66].

Such deleterious effects of Aβ mediated by albumin result paradoxical since in a previous work the protective effect of albumin against toxic effect of Aβ in neurons was informed [34] but here is described that albumin also has the potential to damage neurons, maybe by inducing inflammatory events and/or causing glutamate deregulation. This study suggests that high intraneuronal albumin concentration may be associated with neuronal damage caused by Aβ. This idea would be in coincidence with the increasing evidence which indicates that neurovascular units of BBB are significantly affected in AD in animal models and human patients. Given its high concentration in the plasma, albumin would be expected to gain access to CNS tissue following the breakdown of the BBB. Once in the CNS, albumin has the potential to worsen the disease due to its molecular properties by described mechanisms. Is hypothesized that there may be a threshold determining whether albumin intraneuronal levels are toxic or not. However, further studies are required to demonstrate this hypothesis.

## 5. Conclusions

Formation of Aβ-Albumin complex, which protects neurons against Aβ toxicity, does not require of albumin uptake, supporting that is extracellular. Aβ affects albumin endocytosis preventing late endosomes and lysosomes degradation, probably because of changes in albumin structure or deregulation in vesicular transport. Because of the consequences such as its osmotic effects, the inability to exert its antioxidant properties, its effects on neuronal plasticity and excitability albumin affected endocytosis induced by Aβ is proposed as a new physiopathology mechanism in AD. It is hypothesized that there may be a threshold determining whether albumin intraneuronal levels are toxic or not.
